# Investigation on the Thermodynamic Stability of Nanocrystalline W-Based Alloys: A Combined Theoretical and Experimental Approach

**DOI:** 10.3390/ma14237179

**Published:** 2021-11-25

**Authors:** Francesco Torre, Claudio Mingazzini, Daniele Mirabile Gattia, Teodor Huminiuc, Antonio Rinaldi, Tomas Polcar, Francesco Delogu, Antonio Mario Locci

**Affiliations:** 1 Dipartimento di Ingegneria Meccanica, Chimica e dei Materiali, Università degli Studi di Cagliari, Via Marengo 3, 09123 Cagliari, Italy; torrefrancesco91@gmail.com (F.T.); francesco.delogu@dimcm.unica.it (F.D.); 2 Sustainability Department, SSPT-PROMAS-TEMAF, ENEA, Via Ravegnana, 186, SP302, 48018 Faenza, Italy; claudio.mingazzini@enea.it; 3 Sustainability Department, SSPT-PROMAS-MATPRO, ENEA, Via Anguillarese 301, 00123 Rome, Italy; daniele.mirabile@enea.it (D.M.G.); antonio.rinaldi@enea.it (A.R.); 4 Engineering Materials, Faculty of Engineering and Physical Sciences, University of Southampton, Southampton SO17 1BJ, UK; T.Huminiuc@soton.ac.uk (T.H.); T.Polcar@soton.ac.uk (T.P.); 5 Consorzio Interuniversitario per lo Sviluppo dei Sistemi a Grande Interfase (CSGI), Via della Lastruccia 3, 50019 Sesto Fiorentino, Italy

**Keywords:** nanocrystalline alloys, thermal stability, thermodynamics

## Abstract

The stability of nanostructured metal alloys is currently being extensively investigated, and several mathematical models have been developed to describe the thermodynamics of these systems. However, model capability in terms of thermal stability predictions strongly relies on grain boundary-related parameters that are difficult to measure or estimate accurately. To overcome this limitation, a novel theoretical approach is proposed and adopted in this work to identify W-based nanocrystalline alloys which are potentially able to show thermodynamic stability. A comparison between model outcomes and experimental findings is reported for two selected alloys, namely W-Ag and W-Al. Experimental results clearly highlight that W-Ag mixtures retain a segregated structure on relatively coarse length scales even after prolonged mechanical treatments. Moreover, annealing at moderate temperatures readily induces demixing of the constituent elements. In contrast, homogeneous nanostructured W-Al solid solutions are obtained by ball milling of elemental powders. These alloys show enhanced thermal stability with respect to pure W even at high homologous temperatures. Experimental evidences agree with model predictions for both the investigated systems.

## 1. Introduction

Nanocrystalline (NC) metals exhibit enhanced physical and chemical properties with respect to their bulk counterparts [1,2,3,4,5]. This makes NC metals attractive in many areas of science and engineering. However, their reduced grain size and the consequent high-volume fraction of grain boundaries (GBs) renders NC metals intrinsically unstable and prone to coarsening, such that the properties related to the NC status of the material are eventually lost during fabrication or in-service stages [6,7,8,9,10].

Therefore, in the light of the great promises shown by NC metallic materials in terms of possible applications, research on coarsening-resistant NC metal alloys has been increasingly pursued. This is since alloying has been proved to be the key to a significant improvement of thermal stability of these materials by increasing the temperature range where the as-produced nanostructure can be retained [11,12]. In fact, several NC metallic alloys have shown higher thermal stability with respect to their single-component counterparts. Along these lines, binary [13,14,15,16,17,18,19,20,21,22,23], ternary [24,25,26,27,28,29], and higher order systems have been investigated [30,31,32,33,34].

The enhanced thermal stability of nanostructured alloys may be achieved by acting on the kinetics or the thermodynamics of grain coarsening [35,36]. According to the former approach, a reduction of the GB mobility is obtained through second phases or solutes drag-like effects as well as chemical ordering-induced local rearrangements at GBs [37,38,39]. Alternatively, methods aimed at reducing the coarsening driving force by taking advantage of non-conventional thermodynamic effects induced by GB segregation in metal alloys can also be pursued [40,41]. By comparing the two approaches, it is here worth pointing out that the reduction of GB mobility obtained by the kinetic approach may result into transient stability not persistent to long-term thermal exposures. In addition, the kinetic approach shows the typical Arrhenius-type behavior, while the thermodynamic strategy should be, at least in principle, characterized by a weaker temperature dependence [35]. Thus, it turns out that the thermodynamic approach may be potentially more effective than the kinetic one since nanocrystalline alloys can be designed and developed to maintain their structure at higher temperatures. Moreover, thermodynamic stabilization can provide better control of nanostructures, and increase the number of their processing paths and applications [42,43].

It is then not surprising that since the pioneering work of J. Weissmüller in 1993, several mathematical models or methods have been proposed in the literature to theoretically investigate the thermodynamic stability of nanocrystalline alloys. A detailed description and comparison of various models and approaches can be found in recent reviews [44,45]. However, it should be pointed out that, regardless of the specific model adopted, prediction capability strongly depends on the availability of GB parameters that are usually difficult to measure or estimate reliably. In particular, when the GB phase is described according to the regular solution approximation model the interaction energy in the GB, $\Omega^{\left(gb\right)}$, turns out to be the most critical parameters. As a matter of fact, its value, and consequently its estimation method, strongly influence the theoretical predictions of thermodynamic stability of nanocrystalline materials [46,47]. Unfortunately, broadly accepted and reliable procedures to estimate $\Omega^{\left(gb\right)}$ are not currently available in the literature. Therefore, various evaluation procedures have been followed, while a general and thorough discussion about this topic is still missing.

According to one of the most widespread approaches, the interaction energy $\Omega^{\left(gb\right)}$ is related to interaction energy in bulk, $\Omega^{\left(b\right)}$, as follows:

(1) $$\Omega^{\left(gb\right)}=\beta \Omega^{\left(b\right)}$$

where the coefficient β is regarded as a universal constant [48,49]. A reasonable estimation of this coefficient is based on the ratio between unbroken bonds in the GB region and in the grain interior. For instance, a value of 0.92 was suggested [50]. However, it might also be reasonable that β is related to the specific alloy investigated. Indeed, it should be considered that the interaction between alloy atoms in the GB phase may be diverse from the one that characterize the bulk phase due to three mechanisms. First, a portion or all the elastic strain energy arising from the atomic size mismatch can be released in the GBs. Second, the same alloy constituents may show a different chemical interaction in the GB region with respect to grain interior as an effect of differences in bond energy and atomic coordination number. Finally, the total “defect energy” of the GBs is reduced when atoms with lower GB energy segregate at GBs. Accordingly, the enthalpy of mixing in the GB phase is likely to be significantly different from the one in the bulk state [40,51,52].

This difference may be explored by means of the enthalpy of segregation concept, which is known to reach values up to 100 kJ/mol [53]. The latter quantity was related to the GB interaction energy by Trelewicz and Schuh as follows [53]:

(2) $$\Delta H_{seg}=\left(\Omega^{\left(b\right)}-\Omega^{\left(gb\right)}\right)+\left(\frac{{\hat{v}}_{A}^{0}\sigma_{A}^{\left(gb\right)}-{\hat{v}}_{B}^{0}\sigma_{B}^{\left(gb\right)}}{\zeta^{*}}\right)$$

where $\sigma^{\left(gb\right)}$ and ${\hat{v}}^{0}$ are the GB energy and the molar volume of pure components, respectively, and $\zeta^{*}$ is the GB characteristic thickness. Note that Equation (2) has been derived by setting equals to zero the parameter υ appearing in Equation (25a) of the Trelewicz and Schuh paper; this is for consistency of the model that will be adopted in this work (see next). Rearranging Equation (2), it can be obtained the following expression for the coefficient β:

(3) $$\beta =1-\frac{\Delta H_{seg}}{\Omega^{\left(b\right)}}+\left(\frac{{\hat{v}}_{A}^{0}\sigma_{A}^{\left(gb\right)}-{\hat{v}}_{B}^{0}\sigma_{B}^{\left(gb\right)}}{\zeta^{*}\Omega^{\left(b\right)}}\right)$$

where it can be clearly seen that the assumption of universality for this coefficient is now discarded.

Once that $\Delta H_{seg}$ is known, the interaction energy in the GB phase can be easily obtained through Equation (3). Unfortunately, experimental data of the enthalpy of segregation are scarce and limited to few systems. In addition, their reliability strongly depends on the experimental procedure adopted and the system investigated [54,55,56,57,58,59,60,61] Therefore, it is not surprising that the attempts to relate theoretical predictions to experiments have been typically restricted to alloy systems whose experimental value of $\Delta H_{seg}$ is available or based on fitting procedure to estimate the unknown model parameters. Clearly, these approaches are limited by the availability of experimental information, and thus they cannot provide theoretical predictions of general applicability or be extended to unexplored systems. Then, to overcome the above-mentioned limitations a novel theoretical approach to study the thermodynamic stability of nanocrystalline binary metallic alloys and its experimental validation are presented in this work.

## 2. Materials and Methods

The main goal of the proposed approach is to identify binary metallic systems potentially able to show thermodynamic stability of their nanocrystalline state. This way, the experimental activity can be addressed to alloys with a higher probability of thermally stable nanostructures. The adopted theoretical methods and the experimental methodology implemented for its validation are illustrated and described in Section 2.1 and Section 2.2, respectively.

### 2.1. Theoretical Identification of Thermodynamically Stable Nanocrystalline Alloys

The thermodynamic model of polycrystalline substitutional alloys recently proposed by some of the authors is adopted in this work [46,47]. This model was formulated without the usual limitations to dilute alloys or to immiscible systems. A procedure for the theoretical assessment of thermodynamic stability in polycrystalline alloys by taking advantage of the classical phase equilibria approach and a general criterion for assessing stability against grain growth, phase separation, and intermetallics formation was also proposed. A detailed derivation of the thermodynamic model and examples of its applications can be found in the cited references.

### 2.2. Experimental Assessment of the Thermodynamic Stability of Nanocrystalline Alloys

It was recently suggested that polycrystalline materials could approach their thermodynamically stable (equilibrium) state following two different routes, namely “from below” and “from above” [62]. Specifically, the equilibrium grain size can be eventually attained starting from materials characterized by “finer” or “coarser” structures (cf. Figure 1 of ref. [63]). However, while thermodynamics can take into account both scenarios, experimental evidence of the spontaneous grain refinement representing the “from above” evolution is still lacking. This might be due to the high activation energies for these processes to occur, thus making the thermodynamics-guided refinement hindered by kinetic effects. Moreover, it should be considered that also the “from below” evolution to the material equilibrium grain size may be significantly affected by kinetic barriers [63].

It turns out that an experimental activity aimed to confirm the thermodynamic character of nanoalloy thermal stability may surely be challenging. However, an analysis of experimental results pointed to relate them to kinetic or thermodynamic aspects is prone to be controversial given the present state-of-art of this field. Nonetheless, some conditions could be applied to limit possible misinterpretations of the experimental results [63]. In particular, the starting as-produced materials whose thermal stability is investigated should be characterized by a grain size as fine as possible.

In fact, following this strategy, the thermodynamic stable state (if it exists) could be achieved following the “from below” approach. This is due to the fact that kinetic barriers to grain growth become less effective as the grain size is reduced. Consequently, the existence of a thermodynamically stable structure can be reasonably assumed when steady-state grain size is experimentally observed after prolonged thermal exposure. In fact, the effects of kinetics-related mechanisms on inhibiting grain growth may be excluded in this case since the conditions to facilitate their occurrence were “created”. On the contrary, apparent thermal stability could be experimentally observed when materials with grain size coarser than the equilibrium one is thermally treated. This behavior is due to the combination between the thermodynamically unfavorable grain growth and the kinetically hindered grain refinement (i.e., the “from above” approach). Therefore, the above-described condition is adopted in this work, and the corresponding experimental details are reported in what follows.

#### 2.2.1. Fabrication of Metal Nanoalloys

Nanostructured metallic materials cannot be produced by conventional metallurgical methods [1,2]. To such aim, several innovative methods able to induce far-from-equilibrium conditions has been developed (e.g., vapor deposition to rapid solidification, severe plastic deformation, etc.). However, as already specified, the experimental investigation of thermodynamically stable nanoalloys requires an atomic-scale mixing of elemental species, such as to favor segregation at GBs. Indeed, GB segregation is a spontaneous phenomenon that can be achieved only once the two elemental metals form a homogeneous solid solution.

In light of the considerations mentioned above, mechanical alloying through ball milling (BM) is particularly suitable to the case. Although performed at low temperatures, BM makes two or more elemental metals dispersed so finely to result in their intimate mixing as consequence of the repeated plastic deformation induced to the processed powders [64,65,66]. In this work, a SPEX Mixer/Mill 8000 (SPEX CertiPrep, Metuchen, NJ, USA) was used to fabricate W-Ag and W-Al NC alloys starting from high-purity commercial powders. A batch of 15 g of powders were placed and sealed in a cylindrical jar (height 6.35 cm; diameter 5.71 cm) with three balls (weight 8 g; diameter 0.11 cm) for each run. Resulting ball to powder ratio (BPR) was 1.6. Both hardened steel and tungsten carbide milling media were used in dry conditions. The quantity of powders was assumed to be large enough to make collisions between the milling balls and the internal walls of the vial completely inelastic. Powders were handled by using a glove box with an Ar atmosphere and moisture and oxygen concentration below 5 ppm to avoid contamination due to oxidation processes. Powder processing of increasing duration was then performed under inert atmosphere. Experiments were periodically interrupted to withdraw small amounts of the powder. This allowed monitoring the powders’ structure evolution during BM. Additional details of the adopted experimental procedure can be found elsewhere [67].

#### 2.2.2. Thermal Treatment of Mechanically Alloyed Powders

It was previously mentioned that enhanced coarsening resistance could be understood as an effect of alloying. This means that thermodynamic stability should be ascertained by comparing the thermal behavior of alloys with respect to the pure (commercially) metal counterpart. Therefore, selection of annealing temperatures is a critical aspect that has to be adjusted on the specific system under investigation. Specifically, the melting point of alloy elemental constituents has to be taken into account when assessing the thermal stability. While the case of an alloy of metals with not too different melting points does not present any particular concern, a careful examination should be carried out when the difference in melting point is significant.

In this sense, the W-Al system represents a clear example since the melting point of aluminum is about 2700 °C lower than the tungsten one. Table 1 shows the significant decrease in the melting temperature of body-centered cubic (bcc) W-Al solid solution as the content of Al increases according to the corresponding binary phase diagram [68]. The same table reports homologous temperatures at 700 °C and 900 °C for pure tungsten and selected W-Al alloys. For instance, it can be seen that W and W_80_Al_20_ systems are thermally treated at homologous temperature (Θ) of 0.32 and 0.79, respectively, when annealed at 900 °C. It is then evident that comparing these two systems at the same annealing temperature would make the effect of Al addition on thermal stability affected by diffusion kinetics. Indeed, at that temperature it might be expected that grain growth for pure W is significantly lower than W-Al alloys. Based on these considerations, in this work, the thermal behavior of pure W and W-Al alloys will be compared in terms of homologous temperatures.

According to the considerations discussed above, each system was thermally treated at a homologous temperature in the range 0.1–0.8. However, for some systems, very high or very low temperatures would need to achieve the specified value of Θ. As an example, pure W should have been annealed at 2737 °C to impose Θ = 0.8. Obviously, annealing experiments were not performed in these cases. For a specific value of Θ, samples were thermally treated for different times in the range 30 min to 36 h, to properly evaluate kinetic effects. The metallic powders were exactly weighted in alumina crucibles and heat-treated in a high vacuum furnace under Ar atmosphere. Additional experiments were carried out using a flowing Ar +3% H_2_ mixture.

#### 2.2.3. Characterization

Differential scanning calorimetry (DSC) of powder samples was performed using a Star System TGA/DSC provided by Mettler-Toledo. A small amount (1.3 mg) of powders were inserted in an alumina crucible. A blank measurement was performed by imposing the same heating program to an empty crucible for each run. In this way, instrument and crucible artefacts were opportunely subtracted. A heating rate of 10 °C/min was adopted.

Structural evolution of powders during BM, as well as at the end of annealing experiments, was performed by X-Ray diffraction (XRD) and performed with a SmartLab Rigaku powder diffractometer equipped with a Cu K_α_ source radiation and a monochromator in the diffracted beam. The diffractometer has been operated at 40 kV and 30 mA. XRD patterns have been acquired in the range of 2θ from 20° to 95° with a step size of 0.04 and a time per step of 6 s. The automatic optics and sample alignment routines have been performed to obtain reliable data. XRD patterns were examined according to the Rietveld technique using the Maud software [69]. A SEM-EDS Zeiss LS15 microscopy equipped with a crystal of LaB_6_ as electron source was used to analyze the morphology of BM powders as well as their cross-sections. Micrographs were obtained using a JEOL ARM 200F (STEM) while a FEI Titan3 (TEM and STEM) electron microscopes equipped with a probe and image aberration corrector operating in mode at 200 kV and 300 kV acceleration voltage was adopted for obtaining chemical maps. Latter ones were attained using a Thermo Scientific EDS detector and summed over 100 frames to improve the signal to noise ratio (SNR). Identification of crystal structure and highlighting regions for EDS analysis was performed by taking advantage of selected area electron diffraction (SAED).

## 3. Results and Discussion

### 3.1. Identification of Thermodynamically Stable Nanocrystalline W-Based Alloys

According to the theoretical procedure adopted in this work and described in Section 2.1, the coefficient β defined by Equation (1) is considered a model free parameter [46]. This allows to highlight the central role played by $\Omega^{\left(gb\right)}$ in determining the thermodynamic character of polycrystalline materials stability while avoiding the effects of inaccuracy in the estimation of this parameter. A critical coefficient $\beta^{*}$ is then defined as the threshold value between stability and instability. In our previous work, the critical coefficient $\beta^{*}$ was calculated for all W-based metallic systems [46]. The model results were analyzed by sub-diving the alloys into two groups: (a) alloys with $\Omega^{\left(b\right)}>0$, and (b) alloys with $\Omega^{\left(b\right)}<0$. Model calculations have clearly shown that critical coefficients $\beta^{*}$ are negative for all the alloys of the group (a). Vice versa, the condition $\beta^{*}>1$ (with no exceptions) is obtained for the systems of the group (b). It can be easily demonstrated that the two stability conditions ($\beta <\beta^{*}$ for groups (a) and$\;\beta >\beta^{*}$ for the group (b) can be translated into the condition $\Omega^{\left(gb\right)}<\Omega^{*}\;$. Latter criterion is adopted in this work.

Quantity $\Omega^{*}$ represents the critical value of interaction energy in the GB for thermodynamic stability to take place and it should satisfy the following constraints: $\Omega^{*}<\Omega^{\left(b\right)}$ and $\Omega^{*}<0$. Both conditions are independent of the sign of $\Omega^{\left(b\right)}$ and must be simultaneously fulfilled [46]. A thorough discussion of the significance of these results is reported elsewhere [46,47]. It is here worth recalling that one of the central points of the model adopted in this work is that the GB phase interaction energy, $\Omega^{\left(gb\right)}$, may have different values with respect to the bulk phase one, $\Omega^{\left(b\right)}$. In fact, it is this difference in the mixing enthalpy of bulk and GB phases that can counter-balance the energy penalty of GBs, thus making possible the thermodynamic stabilization of nanostructures.

Predicting whether a given alloy is thermodynamically stable or not relies on the knowledge of both $\Omega^{*}$ and $\Omega^{\left(gb\right)}$. While the value of $\Omega^{*}$ for each specific alloy can be calculated according to the reported procedure, $\Omega^{\left(gb\right)}$ should be necessarily evaluated by other methods. However, as already mentioned, widely accepted and reliable estimation methods for $\Omega^{\left(gb\right)}$ are not reported in the literature.

**Figure 1:**
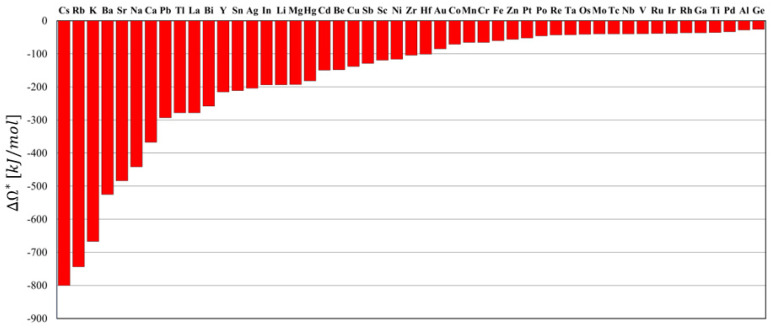
Value of the critical interaction energy difference $\Delta \Omega^{*}$ of W-based alloys.

In this work, a different approach is proposed to bypass this difficulty. Let us take as an example the W-Ag system, whose values of $\beta^{*}$ and $\Omega^{\left(b\right)}$ are −0.16 and 174 kJ/mol, respectively [46]. According to Equation (1), we have that $\Omega^{*}=-29.6\;\mathrm{kJ}/\mathrm{mol}$. We can now define the quantity $\Delta \Omega^{*}=\Omega^{*}-\Omega^{\left(b\right)}$, as the minimum difference between the interaction energy in the grain interior and the one in the GB phase in order to have thermodynamic stability. Then, in the case of the W-Ag system, $\Delta \Omega^{*}=-278\;\mathrm{kJ}/\mathrm{mol}$ is obtained. This calculation was performed for several W-based alloys. A comparative study of various binary systems is shown in Figure 1, where alloys are sorted out in decreasing order of $\Delta \Omega^{*}$.

It can be clearly seen that $\Delta \Omega^{*}<0$ for all the investigated alloys consistently with the stability criteria we have illustrated previously. However, it should also be considered that the difference between the interaction energy in the grain interior and the interaction energy in the GB phase cannot be, realistically, too “large”. After all, we are confronting with the interaction energies of the same atoms pair. Therefore, it might be reasonably assumed that the “probability” for a given alloy to fulfill the stability criteria may be inversely proportional to the magnitude of $\Delta \Omega^{*}$. According to this interpretation, Figure 1 sorts out W-based alloys in terms of a probability for thermodynamic stability to occur. For instance, thermodynamic stability may be predicted “more” probable for nanocrystalline W-Ge alloys ($\Delta \Omega^{*}=-25.7\;\mathrm{kJ}/\mathrm{mol}$) than for W-Cs system ($\Delta \Omega^{*}=-808\;\mathrm{kJ}/\mathrm{mol}$).

With the aim to verify this theoretical approach experimentally, we selected W-Al as a representative of the “more probable” thermodynamically stable nanocrystalline alloys and W-Ag as a representative of the “less probable” ones. Note that the proposed selection took into account also experimental constraints such as alloy powder processability (see Section 2.2).

### 3.2. Experimental Validation of the Theoretical Approach

**Figure 2:**
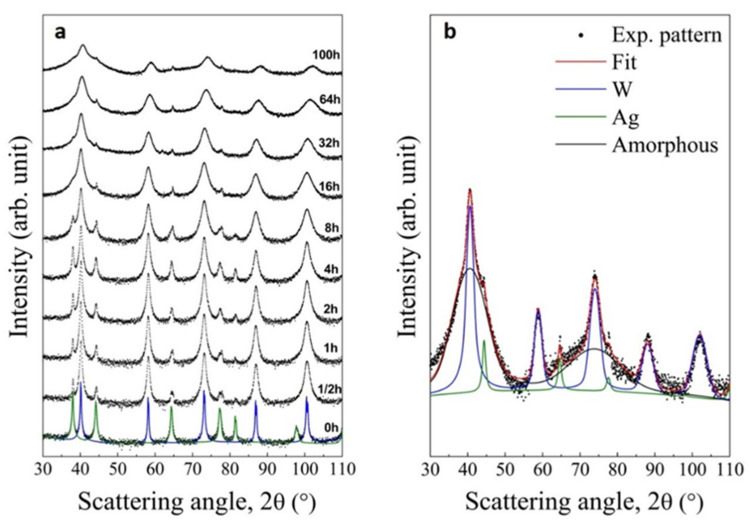
XRD patterns of (**a**) W_85_Ag_15_ powder mixtures at increasing BM times and (**b**) powders processed for 100 h. The presence of different phases is highlight by the best-fitted Rietveld profiles also shown in both figures.

XRD patterns shown in Figure 2a describe the structural evolution of the W_85_Ag_15_ system under the mechanical processing conditions described in the experimental section. Bragg peaks of both bcc W and fcc Ag phases show a progressive broadening, as well as a decrease of the intensity. This indicates the expected microstructural refinement induced by BM [64]. Nevertheless, the complete dissolution of Ag in W lattice were not attained as the Bragg peaks of fcc Ag is still detectable even after 100 h of BM. This can be clearly observed in Figure 2b, where the best fitted profile obtained by the Rietveld method and the contributions of each phase are shown. It can be also observed the presence of an amorphous phase that seems to form after 62 h of BM (cf. Figure 2a). Average grain size of bcc W(Ag) phase after 100 h of BM is about 15 nm while it is about 10 nm for the fcc Ag(W) solid solution. These results agree with those reported for a W-25 wt.% Ag nanostructured composite obtained after 110 h of BM [70].

These results clearly highlight that a single homogeneous W-Ag solid solution cannot be obtained. This finding is likely due to the significantly different mechanical properties of Ag and W. In fact, Ag has a shear modulus of 30 GPa while it is of 160 GPa for W. Similarly, hardness is 0.25 GPa and 3.5 GPa for Ag and W, respectively. Such differences unavoidably induce localized plastic deformation of the Ag phase, while W is almost unaffected. Moreover, thermodynamic factors can intervene Specifically, the very high positive enthalpy of mixing that characterize the W-Ag system (about 40 kJ/mol at the equiatomic composition [71]) can limit the mutual dissolution at the nanometer scale [72,73], thus thermodynamically counter-balancing the forced mixing induced by shearing [74]. Consequently, the mixing process occurring at the atomic scale is significantly hindered in agreement with the behavior shown by systems presenting features similar to the W-Ag one [75,76].

**Figure 3:**
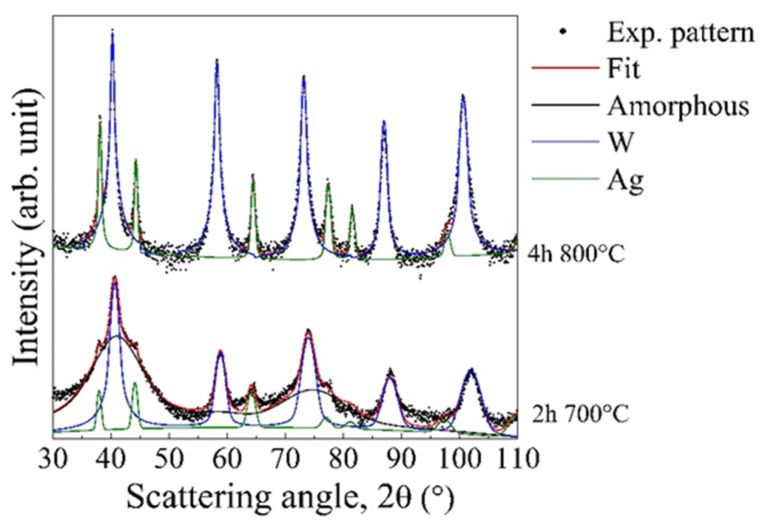
XRD characterization of ball milled (100 h) W_85_Ag_15_ powders and subsequent thermal treatment at 700 °C and at 800 °C for 2 h and for 4 h, respectively.

Ball milled W_85_Ag_15_ powder mixtures processed for 100 h were annealed to verify the nanostructure thermal stability. It is shown in Figure 3 that fcc Ag separated after annealing at relatively low temperatures. In fact, XRD patterns clearly highlight that even short treatments at 700 °C makes Bragg peaks of fcc Ag phase appear. In addition, a significant reduction of the amorphous fraction can be also observed. The same phase separation behavior is further intensified at a higher temperature (800 °C) for a longer annealing time (4 h).

**Figure 4:**
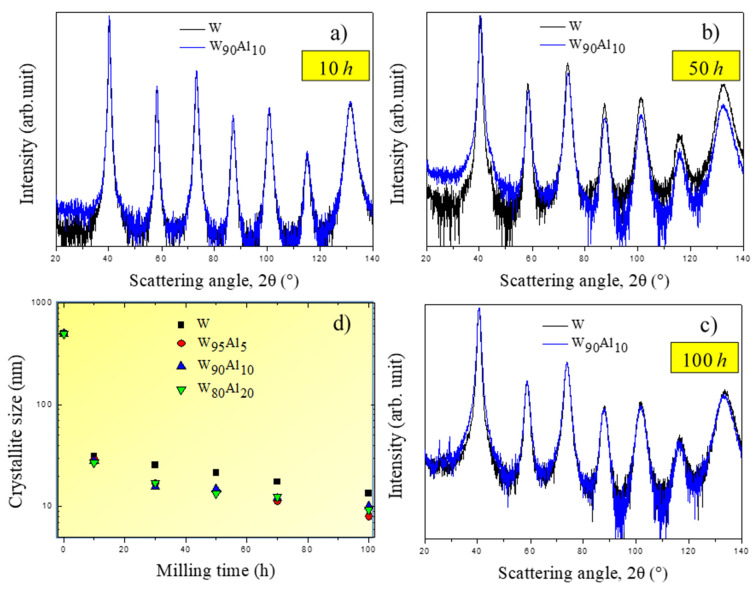
XRD pattern of mechanically treated W_90_Al_10_ mixtures for different ball milling time: (**a**) 10 h; (**b**) 50 h; (**c**)100 h. (**d**) Crystallite size as a function of ball milling time.

The structural evolution of W-Al powder alloys as a function of mechanical treatment time was also investigated by XRD. Patterns of the W_90_Al_10_ alloy are reported in Figure 4a–c for different BM times. It can be seen that only peaks of the bcc W phase are detected, while fcc Al peaks totally disappeared during early stages of BM (cf. Figure 4a). Moreover, although expected according to the phase diagram of the W-Al phase diagram [68], intermetallics formation, i.e., WAl_4_, was not detected by XRD. Bcc W phase peak intensity shows a progressive broadening as an effect of the mechanical treatment. A similar behavior during ball milling processing was shown by the other W-Al alloys taken into account in this work and previously investigated by the authors [67]. Peak broadening reported in Figure 4a–c indicates that mechanical treatment induces a significant structural refinement, as proved by the average crystallite size evaluated with the Rietveld method and shown in Figure 4d. It can be clearly seen that the average crystallite size progressively diminishes and stabilizes at a value of about 10–15 nm for all alloy compositions.

A preliminary study of the thermal stability of W(Al) solid solutions obtained by BM was previously performed by the Authors [67]. In particular, no evidence of Al melting was detected during DSC analysis. This finding suggested that elemental Al is not present in the BM powders, thus indicating that Al may be completely dissolved in the W lattice. In addition, since exothermic peaks were not experimentally observed during DSC, the possible formation of intermetallics was ruled out. For the sake of comparison and confirmation of the latter finding, unalloyed commercial powders of the starting metals were mixed with an 80:20 molar ratio and analyzed by DSC [67]. As expected, Al melting is readily detected and marked by its characteristic endothermic peak at 660 °C. Interestingly, the melting peak of aluminum was immediately followed by an exothermal event occurring in the temperature interval 670–735 °C. As reported in the literature [77], the exothermic peak was related to the intermetallic phases formation. The just mentioned interpretation of DSC results was confirmed by XRD analysis of the thermally treated commercial powder mixture. Specifically, the formation of different intermetallic compounds was observed. In contrast, the XRD pattern of NC W_80_Al_20_ alloys produced by BM and thermally treated under the same conditions showed only peaks of the bcc W(Al) solid solution [67].

All the results reported above clearly indicate that the supersaturated W(Al) solid solutions fabricated by BM are thermally stable with respect to the formation of intermetallics. While kinetic obstacles cannot be completely excluded, it will be shown in the next that the observed stability of W-Al alloy can be explained according to a thermodynamic perspective. It is also worth remarking that both nanocrystalline W_80_Al_20_ and W_90_Al_10_ alloys thermally treated at the above specified conditions showed average crystallite size of about 70 and 60 nm, respectively [67]. In contrast, pure NC W fabricated following the same BM and thermal treatment procedures undergoes rapid grain growth thus reaching an average grain size close to 2 μm. These results indicate a beneficial effect of Al alloying on the thermal stability of W-based nanostructured alloys.

**Figure 5:**
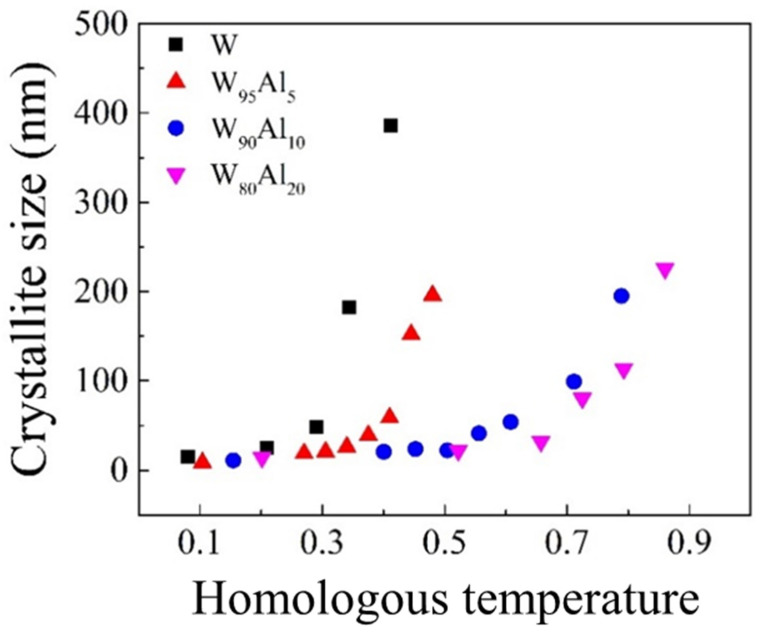
Equilibrium grain size of different W-Al alloys as a function of homologous temperatures. Values obtained for pure W are also included for comparison.

Therefore, in this work thermal behavior of NC W-Al alloys was further studied through a kinetic investigation of grain growth at a constant temperature according to the methodology illustrated in Section 2.2. Results are shown in Figure 5, where the equilibrium crystallite size attained after annealing the BM powders for increasing times is plotted for different W-Al alloys as a function of the homologous temperature. Time required to reach a plateau condition varied from 12 to 36 h depending on the temperature and on the composition. It can be observed that elemental nanostructured W appears stable for low homologous temperatures Θ, while an evident grain growth takes place up to about 400 nm when Θ is increased from 0.3 to about 0.4. Better performances are shown by the W_95_Al_5_ alloy. In fact, its nanostructure is retained up to the homologous temperature of about 0.4. Similarly, W_90_Al_10_ and W_80_Al_20_ systems exhibit significant stability up to homologous temperatures of 0.6 and 0.7, respectively, thus confirming a beneficial effect of Al alloying in terms of coarsening resistance.

**Figure 6:**
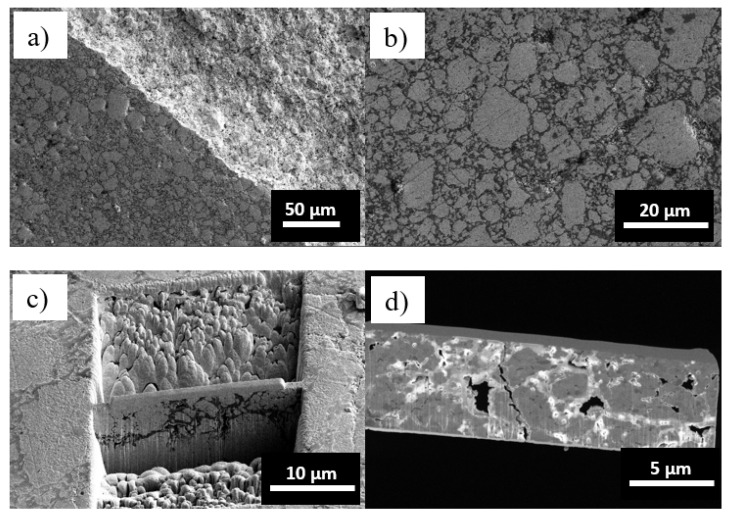
SEM micrographs of W_80_Al_20_ powders after 12 h of annealing at 900 °C. (**a**) Cross-section of a fractured area; (**b**) A detail of the microstructure highlighting partial sintering; (**c**) Preparation of the TEM lamella (**d**).

To obtain further insights on the role played by aluminum alloying, NC W_80_Al_20_ powders annealed at 900 °C for 12 h were analyzed by SEM and TEM. Powders processed with WC milling media were used for this experiment to reduce as much as possible the contamination sources, i.e., Fe from steel media. The prolonged annealing at 900 °C induced the occasional sintering of W_80_Al_20_ powders, as can be seen in Figure 6 where SEM micrographs are reported. This resulted in an irregular and highly porous pellet with a high tendency to fracture (cf. Figure 6a). Particle agglomerates with size ranging from a few hundreds of nm to few tens of μm and characterized by an irregular morphology typical of ball milling are still clearly visible (cf. Figure 6b). In order to perform TEM analysis, a focus ion beam (FIB) was used to obtain a lamella (Figure 6c,d).

**Figure 7:**
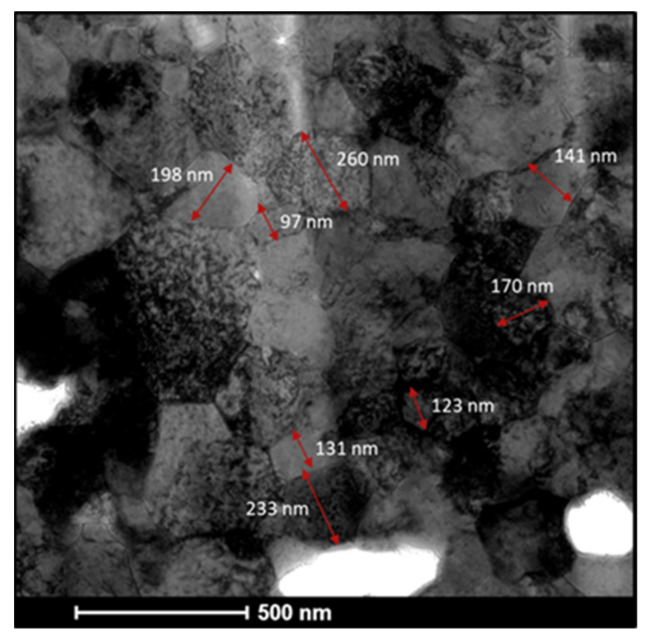
TEM micrograph of W80Al20 powders annealed at 900 °C (Θ ≈ 0.8) for 12 h.

**Figure 8:**
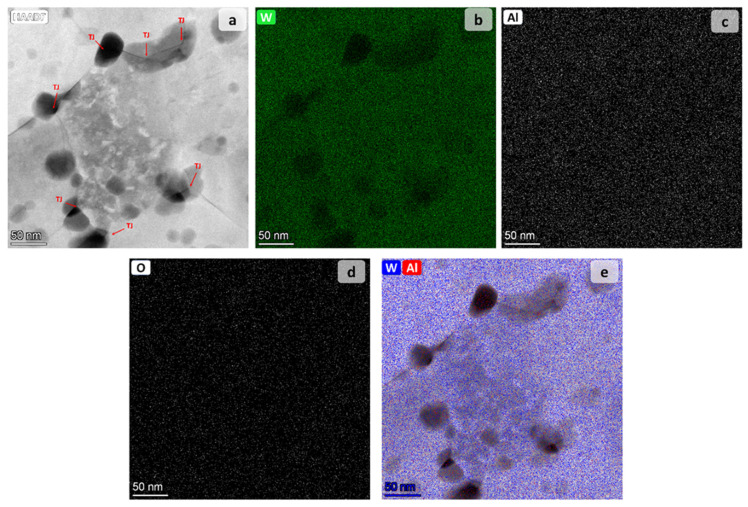
TEM micrograph and chemical mapping of W_80_Al_20_ powders after annealing at 900 °C: (**a**) HAADF image of the microstructure showing darker region which preferentially locate at GBs and triple junctions; chemical distributions of (**b**) W, (**c**) Al and (**d**) O; (**e**) Superimposition of W and Al distribution with HAADR micrograph. Triple junctions (TJ) are indicated by red arrows in Figure (**a**).

A representative particle nanostructure is given by the TEM micrograph reported in Figure 7. An extended analysis of different areas was performed and highlighted a very fine polycrystalline structure with grain sizes ranging from 100 nm up to 250 nm, and with an average grain size of about 170 nm. Such average value is fairly in line with Rietveld estimation (cf. Figure 4). A TEM examination of W_80_Al_20_ annealed powders obtained at higher magnifications is reported in Figure 8, along with the corresponding chemical mapping. A closer look at HAADF micrograph reveals the presence of darker regions, which are preferentially located at GBs and triple junctions (Figure 8a). This finding suggests a tendency of the lighter element (i.e., Al) to segregate. In this regard, EDS analysis of the selected area shows the distribution of W, Al, as well as of O. Latter one shows no preferential location such that the formation of oxides can be ruled out. The elemental distribution confirms a lower concentration of W in the segregated areas at GBs (Figure 8b), where a higher content of Al is instead observed (Figure 8c). This can be better appreciated in the superimposition of the W and Al distributions shown in Figure 8e.

### 3.3. Comparison between Theoretical Predictions and Experiments

The experimental results reported in the previous sections clearly highlight important differences between the behavior of W-Ag and W-Al alloys during ball milling processing as well as in terms of nanostructure thermal stability. W-Ag mixtures retain a segregated structure characterized by a coarse length scales even after prolonged mechanical treatments. Moreover, annealing at moderate temperature readily makes separated W-rich and Ag-rich phases appear. Thus, experimental findings on the W-Ag system agrees with model predictions regarding this system. Indeed, the two-phase (unmixed) state is predicted theoretically as the thermodynamically favored one.

In contrast, homogeneous NC W-Al solid solutions were obtained by BM and their stability after thermal treatment was clearly observed. The nanostructure of W-Al alloys also showed enhanced thermal stability with respect to pure W, even at high homologous temperatures. It should be mentioned that neither the formation of intermetallic compounds nor the precipitation of other secondary phases (e.g., oxides) were observed after the annealing of W-Al alloys. These results seem to rule out the possibility that kinetic hindrances may explain the thermal stability of this system. Vice versa, the latter one may be ascribed to thermodynamic considerations in agreement with model predictions presented in Section 2.

**Figure 9:**
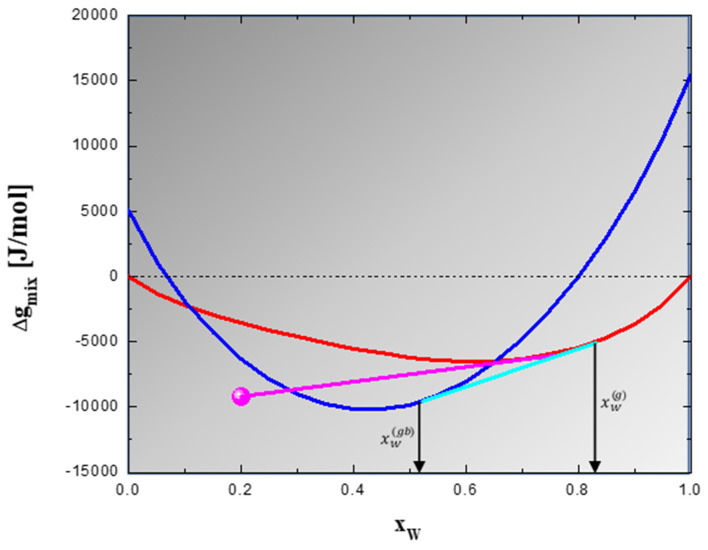
Thermodynamic phase equilibria at 900 °C of polycrystalline of W-Al alloys; Gibbs free energy as function of composition is reported for bulk solid solution (**red**) and GB phase (**blue**). Polycrystalline stable states are given by the common tangent (cyan) between GB phase and bulk solid solution. Purple common tangent describes instead the existence of intermetallic-containing states. The point (**magenta**) identifies the stoichiometric intermetallic compound WAl_4_. The mixing Gibbs free energy of the GB phase has been calculated by using $\Omega^{\left(b\right)}=-14\;\mathrm{kJ}/\mathrm{mol}$ [71] and β=3.82 [46], while the value of WAl_4_ is taken from Ansara et al. [68].

Further insights about the thermodynamic nature of the thermal stability of W-Al alloys can be gained from Figure 9 where phase equilibria at 900 °C are reported according to the model adopted in this work. To shed some light on the thermodynamics of this system, let us focus on the W_80_Al_20_ alloy, which is the one analyzed in Figure 8. In can be clearly seen that the most stable state of this alloy is represented by the polycrystalline structure (cyan tangent line), which is in line with the observed thermal stability. It is also shown in Figure 9 that the system composed of the solid solution and the WAl_4_ phase (purple tangent line) has a higher Gibbs free energy of mixing compared to the polycrystalline state. This finding is in agreement with the experimentally undetected formation of intermetallic compounds.

According to Figure 9, the thermodynamically stable polycrystalline structure is composed of a grain interior and a GB phase with a composition of about $x_{W}^{\left(g\right)}=0.83$ and $x_{W}^{\left(gb\right)}=0.52$, respectively. This implies the segregation of aluminum to form Al-rich regions at the GBs. Although a quantitative evaluation of the composition of the different areas shown in Figure 8 is not available, model predictions agree at least qualitatively with the mentioned experimental results. By applying the lever rule to the model results shown in Figure 9, a volume fraction of the GB, $f^{\left(gb\right)}$, equal to about 0.096, can be calculated. This result is comparable with the fraction of darker areas appearing in Figure 8a.

It should be remarked, however, that the mathematical model adopted in this work does not include the alloy microstructure geometry. Therefore, further comparison between theoretical predictions and the microstructural features shown in Figure 8 should rely on a relationship between grain size and volumetric fractions of the phases that compose the system. For instance, using Equation (5) with the values of $f^{\left(gb\right)}$ reported above and of $d^{\left(g\right)}=150\;\mathrm{nm}$ (cf. Figure 8a), a value of the GB thickness, t, equal to about 5 nm is obtained. While this result appears certainly realistic [78], the experimental counterpart cannot be easily identified in Figure 8. In addition, it should be pointed out that Equation (5) cannot describe the extended darker areas shown in the same figure. This is not surprising since Equation (5) is typically associated to GB segregation in immiscible or partly miscible systems, i.e., $\Omega^{\left(b\right)}>0$, which represents the vast majority of the alloys investigated in thermodynamic stability-related literature [11,79]. Segregation in these systems is strongly localized to few atomic layers close to GBs and larger regions such as the ones shown in Figure 8 are not typically observed [79,80,81].

However, some researchers have also focused on systems that have a negative enthalpy of mixing ($\Omega^{\left(b\right)}<0$) [82]. As an example of this category let us consider the Ni-Zr alloys. This system has some similarities with the W-Al alloy investigated in this work. Indeed, both systems have a negative enthalpy of mixing. Moreover, intermetallic compounds as well as a limited solubility of Zr and Al in fcc Ni and bcc W, respectively, characterizes both bulk phase diagrams. It can then be interesting to also compare the experimental findings for these two systems.

Specifically, a Ni-5.5 at.% Zr alloy prepared by magnetron sputtering and then annealed shows experimental findings very similar to the results obtained in this work for the W-Al system [82]. In particular, an fcc Ni (Zr) solid solution was obtained while other phases (e.g., intermetallics) were not detected. Moreover, the average grain size remains in the nanocrystalline range after thermal treatment, thus showing the coarsening resistance of this alloy. More interesting, EDS mapping of TEM image of the Ni-Zr alloy (cf. Figure 7b of the cited reference) shows a segregation behavior of Zr quite similar to the one of Al revealed in Figure 8.

It is worth adding that the behavior of the Ni-Zr system was predicted according to an empirical rule [82]. Specifically, a positive enthalpy of segregation, $\Delta H_{seg}$, coupled with a negative enthalpy of mixing, $\Delta H_{mix}$*,* should promotes the nanoscale formation of amorphous intergranular films or regions where GB segregation is localized. While the amorphous nature of the segregated areas shown in Figure 8 was not investigated in this work, it may be useful to verify the empirical rule proposed by Schuler and Rupert for the case of W-Al alloys. We already specified that $\Delta H_{mix}<0$ for this system. As far as the enthalpy of segregation is concerned, using Equation (2) and setting the model parameters for the W-Al system, the result $\Delta H_{seg}=44.5\;\mathrm{kJ}/\mathrm{mol}$ is calculated. Therefore, it appears that both theoretical predictions and experimental findings presented in this work for W-Al alloys agree with the results reported in the literature for similar systems.

## 4. Conclusions

A theoretical approach of general application (e.g., without the limitation to immiscible systems or the assumption of dilute solutions) is adopted in this work to identify nanocrystalline metal alloys potentially able to show thermodynamic stability. This way, stability against grain growth, phase separation or intermetallic phase formation can be predicted. Specifically, a critical value $\Omega^{*}$ of the parameter, $\Omega^{\left(gb\right)}$, is obtained such that the relation $\Omega^{\left(gb\right)}<\Omega^{*}$ can be used as a criterion for the assessment of thermodynamic stability. The proposed theoretical tool is applied to perform a comparative analysis of various W-based binary alloys.

Model predictions and experimental findings are compared for two selected systems, namely W-Ag and W-Al. Experimental results clearly highlight marked differences between the behavior of these alloys during ball milling processing as well as in terms of thermal stability. W-Ag mixtures maintains a segregated structure characterized by a relatively coarse length scales even after prolonged mechanical treatments. Moreover, annealing at moderate temperature readily induces the growth of separated W-rich and Ag-rich domains. This experimental evidence on the W-Ag system supports the model outcomes. Indeed, the two-phase (unmixed) state is predicted theoretically as the thermodynamically favored one.

In contrast, homogeneous NC W-Al solid solutions were obtained by BM of elemental powders. As predicted by the adopted theoretical approach, W-Al alloys experimentally showed enhanced thermal stability with respect to pure W even at high homologous temperatures. Moreover, neither the formation of intermetallic compounds nor the precipitation of secondary phases (e.g., oxides) were observed after the annealing of W-Al alloys. Latter findings seem to rule out kinetic contributions to the enhanced thermal stability of these alloys. TEM observation of annealed samples shows the marked tendency of aluminum atoms to segregate at GBs. This finding indicates that solute segregation at GBs may act as a stabilizer of nanostructure also in binary alloys of elements characterized by a negative enthalpy of mixing. Theoretical predictions and experimental findings presented in this work for the case of W-Al system have also been compared with literature results for similar alloys. A substantial agreement in terms of thermal stability of the nanostructures as well as of the GB segregation features was found.

## Figures and Tables

**Table 1:** Melting temperature and homologous temperatures at 700 °C and 900 °C of W and selected W-Al alloys.

	T_m_ [°C]	Θ_700 °C_	Θ_900 °C_
W	3422	0.26	0.32
W–5 at.% Al	2587	0.34	0.41
W–10 at.% Al	1657	0.50	0.61
W–20 at.% Al	1207	0.66	0.79

$\Theta =T/T_{m}$, where $T_{m}$ the solidus temperature of the bcc W(Al) solid solution and T is the annealing temperature.

## Data Availability

Data is contained within the article.
